# Feasibility of generalised diffusion kurtosis imaging approach for brain glioma grading

**DOI:** 10.1007/s00234-020-02613-7

**Published:** 2021-01-07

**Authors:** E. L. Pogosbekian, I. N. Pronin, N. E. Zakharova, A. I. Batalov, A. M. Turkin, T. A. Konakova, I. I. Maximov

**Affiliations:** 1 Neuroimaging Department, N.N. Burdenko National Medical Research Centre of Neurosurgery, Moscow, Russian Federation; 2 General and Clinical Neurophysiology Lab, Institute of Higher Nervous Activity and Neurophysiology of RAS, Moscow, Russian Federation; 3 Department of Psychology, University of Oslo, Oslo, Norway; 4 Department of Mental Health and Addiction, Norwegian Centre for Mental Disorders Research (NORMENT), Oslo University Hospital, Oslo, Norway; 5 Department of Health and Functioning, Western Norway University of Applied Sciences, Bergen, Norway

**Keywords:** Glioblastoma, Glioma discrimination, Diffusion kurtosis imaging

## Abstract

**Purpose:**

An accurate differentiation of brain glioma grade constitutes an important clinical issue. Powerful non-invasive approach based on diffusion MRI has already demonstrated its feasibility in glioma grade stratification. However, the conventional diffusion tensor (DTI) and kurtosis imaging (DKI) demonstrated moderate sensitivity and performance in glioma grading. In the present work, we apply generalised DKI (gDKI) approach in order to assess its diagnostic accuracy and potential application in glioma grading.

**Methods:**

Diffusion scalar metrics were obtained from 50 patients with different glioma grades confirmed by histological tests following biopsy or surgery. All patients were divided into two groups with low- and high-grade gliomas as grade II versus grades III and IV, respectively. For a comparison, trained radiologists segmented the brain tissue into three regions with solid tumour, oedema, and normal appearing white matter. For each region, we estimated the conventional and gDKI metrics including DTI maps.

**Results:**

We found high correlations between DKI and gDKI metrics in high-grade glioma. Further, gDKI metrics enabled introduction of a complementary measure for glioma differentiation based on correlations between the conventional and generalised approaches. Both conventional and generalised DKI metrics showed quantitative maps of tumour heterogeneity and oedema behaviour. gDKI approach demonstrated largely similar sensitivity and specificity in low-high glioma differentiation as in the case of conventional DKI method.

**Conclusion:**

The generalised diffusion kurtosis imaging enables differentiation of low- and high-grade gliomas at the same level as the conventional DKI. Additionally, gDKI exhibited higher sensitivity to tumour heterogeneity and tissue contrast between tumour and healthy tissue and, thus, may contribute as a complementary source of information on tumour differentiation.

## Introduction

Primary brain glioma is a highly widespread type of intra-axial brain tumours seen approximately in one-fourth diagnosed tumour cases [1]. Classification of the brain tumours is performed in accordance with grades introduced by the World Health Organization (WHO) [2] which combine histopathological and molecular features into integrated tumour characterisation. As a result, glioma grades are ordered from grades I up to IV related to tumour’s aggressiveness and malignancy. Both the tumour’s diagnosis and its grade can be reliably confirmed by a histopathologic analysis of the tumour’s tissue obtained from an invasive procedure such as biopsy or surgery. However, non-invasive assessments of the tumour’s malignancy are necessary for clinical treatment, surgery planning, and survival rate estimations. In particular, this applies to low-grade tumour cases where such assessments contribute to treatment efficacy and, as a result, improve quality of life for patients. Magnetic resonance imaging (MRI) offers a wide spectrum of tissue visualisation approaches for clinical purposes allowing one to detect and localise brain’s abnormalities with a certain degree of specificity. Nevertheless, conventional MRI techniques such as structural *T*_1_/*T*_2_-weighted imaging with or without contrast agents or magnetic resonance spectroscopy demonstrate limited sensitivity and specificity for brain glioma differentiation [3, 4].

Diffusion-weighted imaging (DWI) has exhibited its superiority as a non-invasive brain imaging technique and has been applied in a plethora of clinical settings [5–7]. Diffusion tensor imaging (DTI) [8], the most often used DWI approach, has also been utilised for tumour assessment by many research groups [9–11]. In turn, diffusion kurtosis imaging (DKI) [12] is a powerful extension of the conventional DTI technique enabling estimation of the degree of non-Gaussian diffusion in the brain tissue by estimating higher order cumulant of the diffusion signal expansion [13]. The deviation of water molecule diffusion from free diffusion behaviour or Gaussian distribution is caused by complex organisation of the brain tissue, where cellular and neurite barriers alter the probability distribution of water diffusion. This phenomenon is particularly important for the brain tumour detection and assessment due to increased tissue complexity resulting from the high cancer cell proliferation rate, increased tumour tissue vascularisation, presence of oedema, as well as necrosis. DKI is extensively used in brain imaging [14–16], in particular, for tumour differentiation [17–21]. Despite the fact that DKI demonstrated quite promising results in the case of glioma grading, the kurtosis scalar metrics, such as mean kurtosis, still lack the accurate and reliable glioma grading in the case of both low- (i.e. glioma-I and II) and high- (glioma-III and IV) grade gliomas [18, 22, 23].

In the present study, we adapted the generalised kurtosis approach (GK) [24] for glioma differentiation in order to evaluate as well as to add a complementary information and, thus, increase the accuracy of glioma grading. An advantage of the third-order term of cumulant expansion of the diffusion signal has been demonstrated for differentiation of low- and high-grade gliomas together with an application of the conventional kurtosis (CK) approach. In order to emphasise the advantage of the DKI imaging for glioma grading, we concurrently applied both DKI and DTI metrics derived from the CK and GK approaches. Hence, the purpose of the present study was to investigate the value of the generalised DKI metrics for the evaluation of glioma grading.

## Materials and methods

### Patients

Fifty adult patients (mean age, 44 ± 13 years; minimal age, 24 years; maximal age, 70 years; male/female ratio, 30/20) initially diagnosed with glioma were recruited at our Institution and examined with MRI prior to biopsy, surgery, radiation or chemotherapy treatments. The diagnosis of glioma and the WHO grade [25] were confirmed by histological and immunohistochemical examinations for each subject following MRI examination. Complementary to the histological glioma grade evaluation, the immunohistochemical marker Ki-67 was extracted from pathology using commercial monoclonal antibody. Immunohistochemistry of Ki-67 was performed and retrospectively analysed by a pathologist blinded to the clinical- and MRI-derived information. The Ki-67 rates were estimated by the percentile of immunoreactive cells from 1000 malignant cells. The estimation was performed in the areas with the highest number of positive nuclei within the solid tumour.

The study included 19 patients with glioma grade II, 8 patients with glioma grade III, and 23 patients with glioma grade IV. The group of patients with glioma-II consisted of 6 subjects with oligoastrocytoma, 1 subject with oligodendroglioma, 1 subject with gemistocytic astrocytoma, and 11 subjects with diffuse astrocytoma. The group of patients with glioma-III consisted of 7 subjects with anaplastic astrocytoma and 1 subject with anaplastic oligoastrocytoma. The group of patients with glioma-IV consisted of the 22 subjects with glioblastoma and 1 subject with gliosarcoma.

### Imaging

All patients underwent MRI examination with a 3T GE scanner (Signa HDxt) using an 8-channel phased-array head coil. The imaging protocol included precontrast *T*_2_-weighted FLAIR and diffusion-weighted sequences, followed by postcontrast *T*_1_-weighted imaging with a gadolinium-based contrast agent. The parameters specific to anatomical imaging sequences were the following: *T*_1_-weighted: FOV = 240 mm^2^, TE/TR = 3500/8800 ms, resolution 1 × 1 × 1 mm^3^; *T*_2_-weighted FLAIR: FOV = 240 mm^2^, TE/TR = 120/9500 ms, resolution 1 × 1 × 5 mm^3^; diffusion-weighted imaging: FOV = 240 mm^2^, TE/TR = 102.8/10000 ms, resolution 3 × 3 × 3 mm^3^, *b* values = 0, 1000, and 2500 s/mm^2^ with 60 encoding diffusion directions per each *b*-shell; directions were non-coplanar uniformly distributed over the unit sphere. The diffusion imaging was obtained using a single-shot echo planar imaging sequence.

### Image analysis

**Fig. 1:**
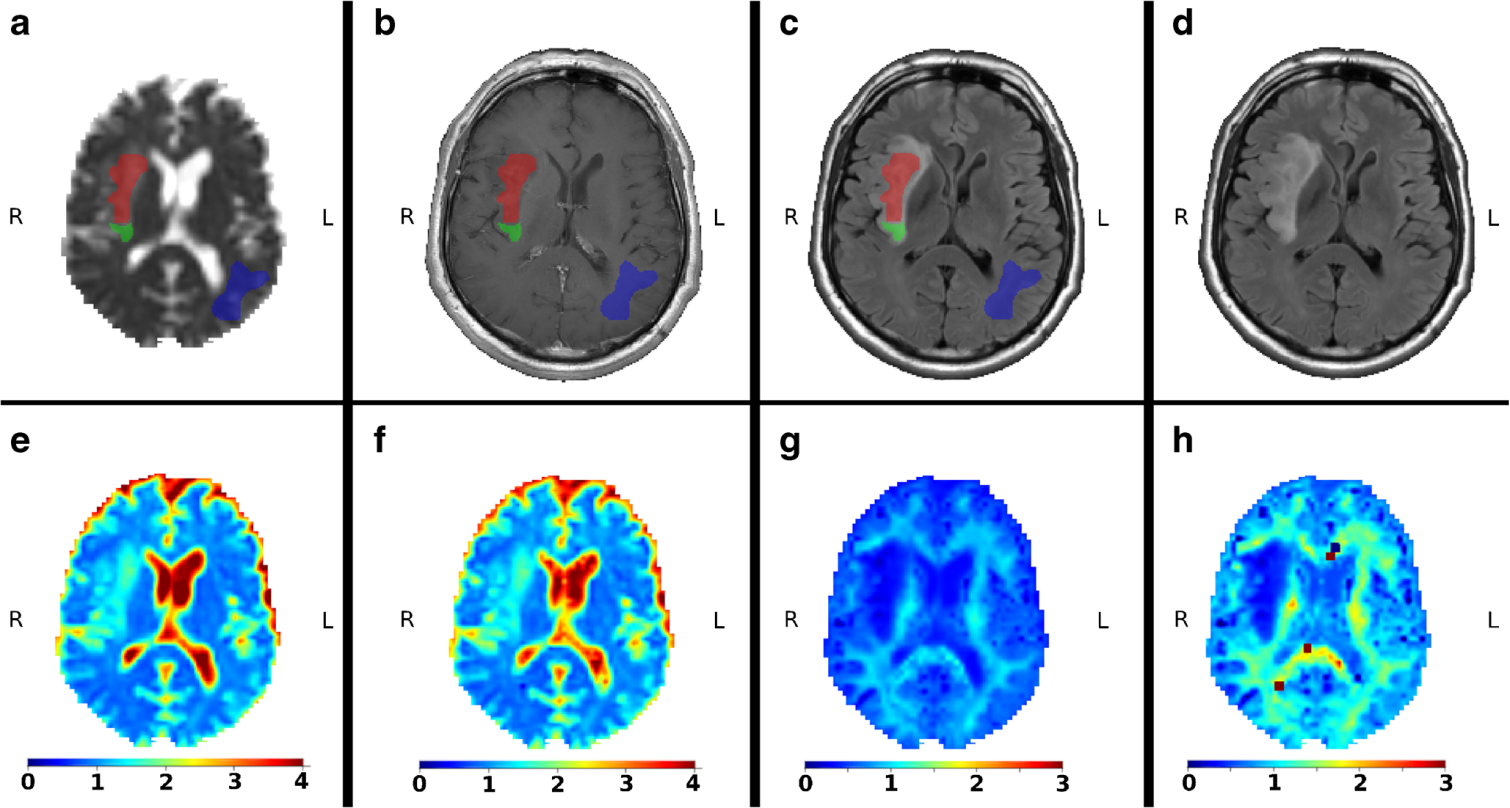
Example of low-grade gliomas with the marked regions of interests: red is the tumour, green is the oedema, and blue is the NAWM. All maps are already aligned and interpolated to *T*_1_-weighted image. **a** mean diffusivity map with masks; **b** *T*_1_-weighted image with contrast agent; **c** *T*_2_-weighted FLAIR image with contrast agent; **d** *T*_2_-weighted FLAIR image without contrast agent; **e** mean diffusivity map obtained from the conventional kurtosis approach; **f** mean diffusivity map obtained from the generalised kurtosis approach; **g** mean kurtosis map obtained from the conventional kurtosis approach; and **h** mean kurtosis map obtained from the generalised kurtosis approach

**Fig. 2:**
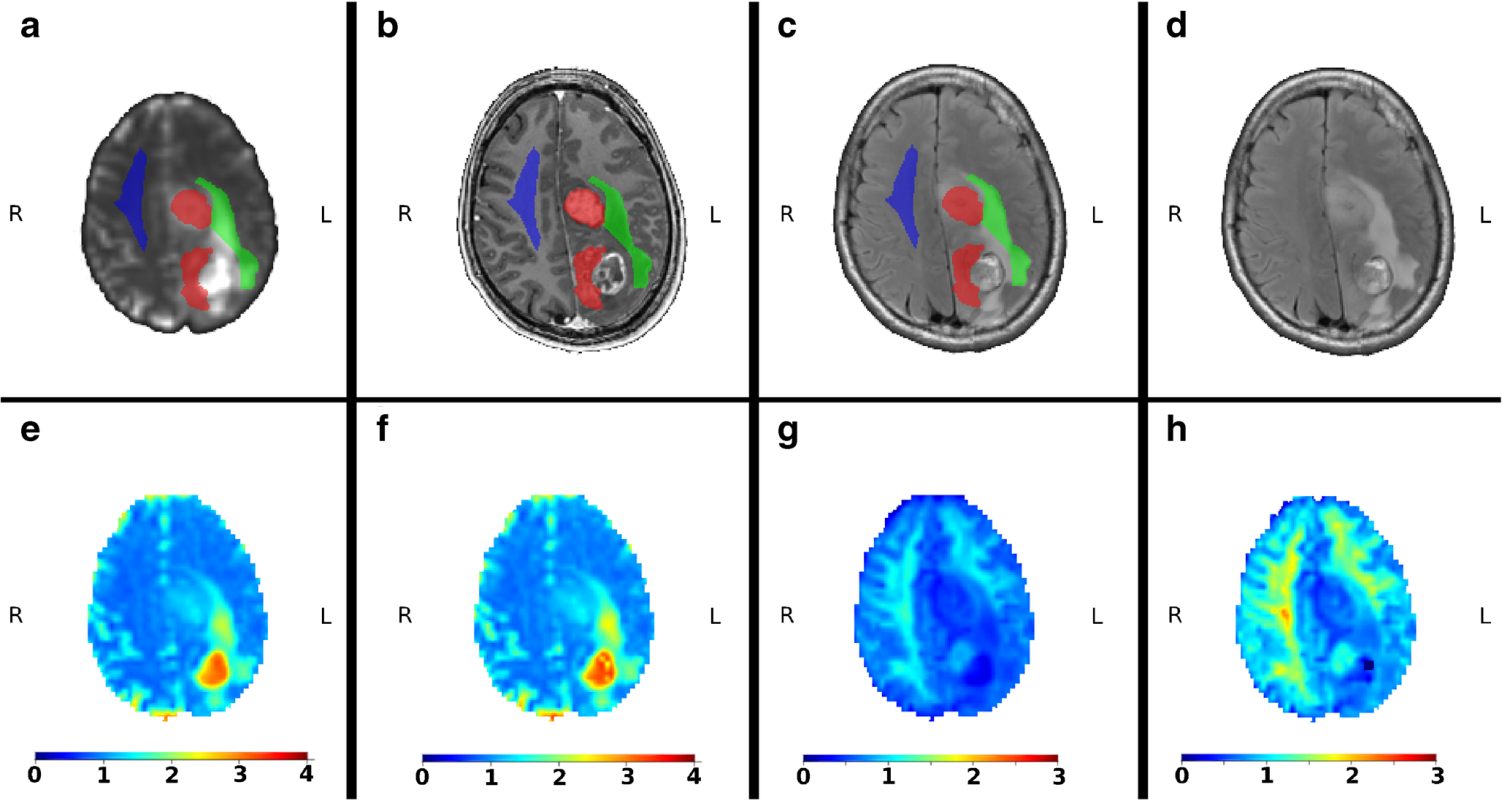
Example of high-grade gliomas with the marked regions of interests: red is the tumour, green is the oedema, and blue is the NAWM. All maps are already aligned and interpolated to *T*_1_-weighted image. **a** mean diffusivity map with masks; **b** *T*_1_-weighted image with contrast agent; **c** *T*_2_-weighted FLAIR image with contrast agent; **d** *T*_2_-weighted FLAIR image without contrast agent; **e** mean diffusivity map obtained from the conventional kurtosis approach; **f** mean diffusivity map obtained from the generalised kurtosis approach; **g** mean kurtosis map obtained from the conventional kurtosis approach; and **h** mean kurtosis map obtained from the generalised kurtosis approach

Prior to estimation of scalar diffusion metrics, the raw diffusion data were corrected for image distortions in accordance with optimised pipeline [26], i.e. for noise [27] and Gibbs-ringing distortions [28], eddy-current distortions including bulk head motion artefacts were corrected using *eddy* utility from FSL [29–31]. Conventional DKI metrics such as mean, axial, radial kurtosis, and kurtosis anisotropy (MK, AK, RK, and KA, respectively) were estimated using an iterative weighted least squares approach [32] and supplied by the complementary DTI measures known as fraction anisotropy (FA), mean diffusivity (MD), axial diffusivity (AD), and radial diffusivity (RD). The conventional DKI metrics were obtained from the second-order cumulant expansion in the following form [12]:

$$ \ln \left[S(b)\right]=\ln \left[{S}_0\right]-\mathrm{bD}+{\mathrm{b}}^2{D}^2\frac{K}{6}={\mathrm{F}}_{1,} $$

where *b* is the diffusion weighting, *D* is the diffusion coefficient, *S*_0_ is the signal without the diffusion weightings, and *K* is the diffusion kurtosis. The right-hand expression is defined as *F*_1_. The generalised DKI parameters were estimated using in-house MATLAB scripts (The MathWorks, Natick, MA, USA) including the third-order terms [24]:

$$ \ln \left[S\left(\mathrm{b},\upalpha \right)\right]={\mathrm{F}}_1+\left(\alpha -1\right)\cdotp {D}^3{b}^3\frac{K^2}{54}, $$

where *α* is the adjusting variable. In the case of *α* = 1, the last equation leads to the conventional DKI expression *F*_1_. In the present work, we used an optimised *α* value at 2/7 [24]. Subsequently, the estimated diffusion scalar maps for both cases of conventional and generalised kurtosis were aligned and interpolated to *T*_1_-weighted anatomical image for each patient using the cubic spline interpolation algorithm and affine transformation. Two independent radiologists manually delineated three regions of interest for each subject: tumour, oedema, and normal appearing white matter (NAWM) using 3 MRI contrasts: *T*_1_-weighted, *T*_2_-weighted FLAIR, and MD map estimated from CK approach. Examples of images for low- and high-grade gliomas with the 3 aforementioned regions of interest are presented in Figs. 1 and 2, respectively.

### Statistical analysis

Statistical analyses were performed using *R* Project (http://r-project.org) and in-house MATLAB scripts. Some voxels among the patient maps contained of corrupted diffusion metrics derived from the DTI and DKI tensors. In order to prevent any statistical bias in analysis, we excluded such voxels from consideration using inequalities: MK < 0 and MK > 3, for both CK and GK approaches. Mean values and standard deviations of all diffusion metrics were calculated from the tumour, oedema, and NAWM regions. These values were used for the low- and high-grade glioma differentiation and assessed with a Mann-Whitney-Wilcox *U* test with statistical significance reported at the 0.05 level. Effect sizes were calculated with Cohen’s *d* in order to estimate the magnitude of the differences between the conditions/groups. Correlations between the conventional and generalised DKI metrics have been estimated using a linear regression model: *y* = *k*_0_ + *k*_1_•*x* and package *stat* from *R* Project. Receiver operating characteristic (ROC) analysis was performed using package *pROC* in order to compute the area under curve (AUC) for estimation of the diffusion model performance for tumour differentiation. The squared Pearson correlation coefficients were estimated using the package *stat* from *R* Project. In order to estimate a rate of the solid tumour heterogeneity using diffusion metrics derived from CK and GK approaches, we used a root-mean-square estimator (RMSE). The RMSE rate demonstrates the root-mean-square deviation of the diffusion metric from the tumour mean value averaged over the tumour region, i.e. the higher RMSE rate exhibits a higher metric dispersion or heterogeneity.

## Results

Figures 1 and 2 are visual examples of manual masks of three regions, i.e. tumour, oedema, and NAWM together with examples of MD and MK maps for the low- and high-grade glioma patients, estimated by the CK and GK approaches. All maps are presented in *T*_1_-weighted space with a spatial interpolation of diffusion maps. Briefly, we can see that the visual quality of MD maps between CK and GK approaches is very similar. In turn, as already was shown in [24], the tissue contrast for DKI maps, e.g. MK, is higher for GK approach. Squared outlier voxels both in Figs. 1 and 2 are the result of fitting problems due to noisy diffusion signal. These voxels have been excluded from the analysis.

**Fig. 3:**
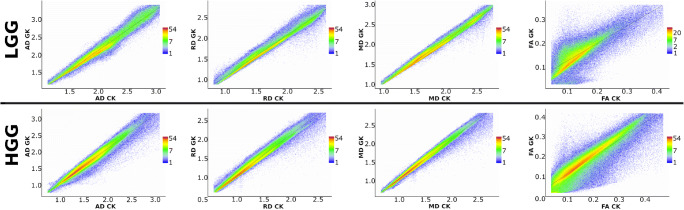
Voxel-wise correlations of the diffusion tensor metrics between the conventional and generalised kurtosis approaches. The scalar metrics belong to the tumour masks in low- and high-grade gliomas for all patients. The columns of plot are axial, radial, and mean diffusivities and fractional anisotropy, respectively

**Fig. 4:**
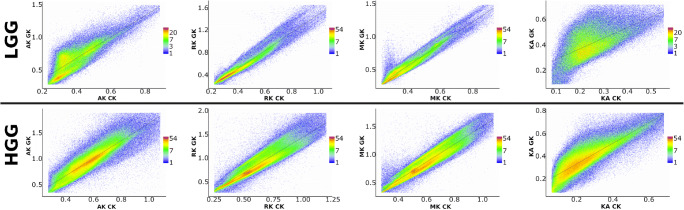
Voxel-wise correlations of the diffusion kurtosis metrics between the conventional and generalised kurtosis approaches. The scalar metrics belong to the tumour masks in low- and high-grade gliomas for all patients. The columns of plot are axial, radial, and mean kurtosis and kurtosis anisotropy, respectively

Figures 3 and 4 present the correlations of diffusion metrics obtained by CK and GK signal expansions. Figure 3 shows DTI metrics only, i.e. FA, MD, AD, and RD, with corresponding R-squared values which in the case of linear correlations coincide with the squared Pearson correlation coefficients (see Table 1). In Fig. 4, we present DKI metrics only, i.e. KA, MK, AK, and RK, with corresponding R-squared values (see Table 1). The scatter plots were estimated voxel-wisely for the tumour masks only from low- and high-grade gliomas, respectively. In short, linear correlations between CK and GK estimations are higher for the high-grade glioma in contrast to the low-grade glioma. This effect is present for all DTI metrics. In the case of DKI metrics, it applies only for KA and AK metrics. Interestingly, MK and RK metrics have two linearly dependent subgroups of voxels (see, e.g. Fig. 4: LGG), which merge as glioma malignancy increases (see, Fig. 4: HGG).

**Table 1:** The results for linear correlations between diffusion metrics obtained from CK and GK approaches (see Figs. 3 and 4)

	FA	MD	AD	RD	KA	MK	AK	RK
LGG								
Intercept *b*_0_	0.053	−0.017	0.091	0.025	0.19	−0.12	0.0088	−0.15
Slope *b*_1_	0.73	1.1	1.0	1.0	0.88	1.7	1.7	1.6
*R*-square	0.693	0.981	0.906	0.97	0.463	0.944	0.777	0.942
HGG								
Intercept *b*_0_	0.028	0.022	0.054	0.028	0.14	−0.1	−0.018	−0.13
Slope *b*_1_	0.85	1.0	1.0	1.0	0.93	1.6	1.7	1.6
*R*-square	0.814	0.977	0.953	0.97	0.756	0.889	0.804	0.855

The linear model is *y* = *b*_0_ + *b*_1_∙*x*; R-square is the coefficient of determination also coinciding with the squared Pearson correlation coefficient. *LGG* low-grade glioma, *HGG* high-grade glioma

**Fig. 5:**
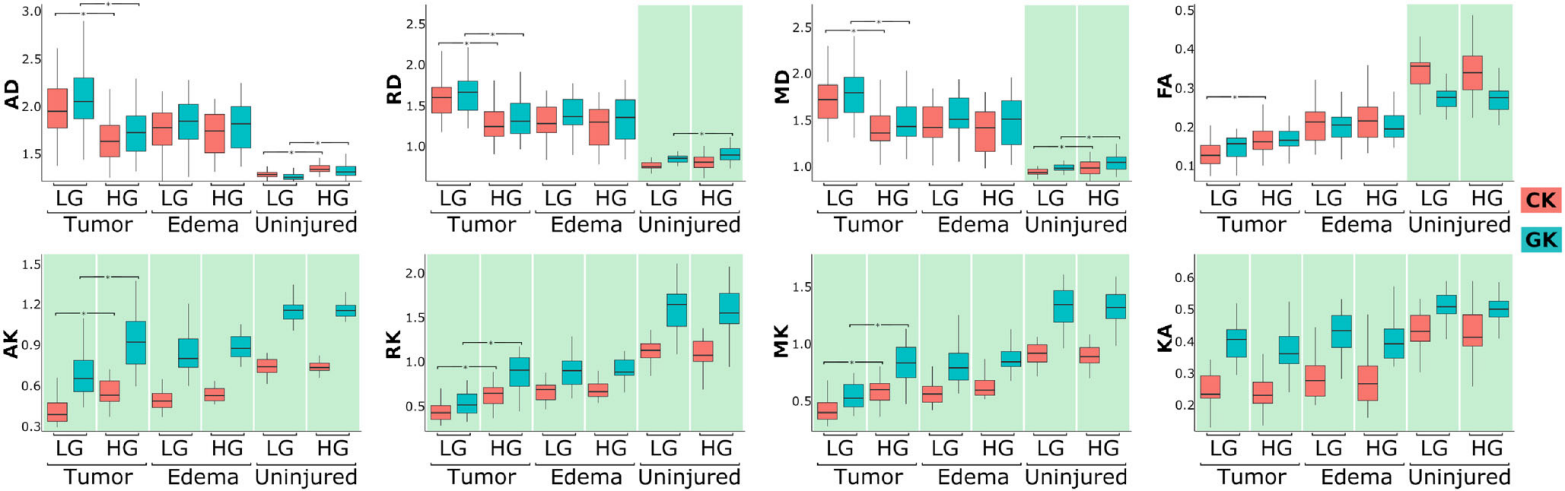
The boxplots of averaged diffusion tensor and kurtosis metrics in different regions: tumour, oedema, and normally appeared white matter. The star-marked brackets exhibit the significant (*p* < 0.05) difference between low- and high-grade gliomas for the diffusion metrics. The green background colour emphasise the significant difference between diffusion metrics for the conventional and generalised approaches

In Fig. 5 we present boxplots of the mask-averaged diffusion metrics estimated from CK and GK approaches. The averages were performed for each ROI such as tumour, oedema, and NAWM for each patient. The boxplots are structured as ROI-wise and glioma grades pairs. For each pair of low- and high-grade gliomas, we performed the Mann-Whitney-Wilcox *U* test in order to check for significant (*p* < 0.05) differences between glioma grades in all regions including tumour, oedema, and NAWM. There were no significant differences between low- and high-grade glioma diffusion metrics for oedema, and there was difference between GK metrics of RD for NAWM. However, for tumour regions, both CK and GK metrics except for KA and FA, we revealed a significant difference for low- and high-grade gliomas (marked by asterisk in Fig. 5). In the case of FA metrics, GK approach did not reveal significant differences between glioma grades. The effect sizes for the diffusion metric differences detected by both CK and GK approaches are summarised in Table 2. For DTI metrics, the effect size is large for both kurtosis approaches, while for DKI metrics the differentiation possesses very large effect size except for GK-derived RK metric. We also performed a pair comparison between CK and GK metrics marked by the cyan background colour for significant differences (*p* < 0.05) between the CK-GK metrics. Interestingly, for all DKI metrics and ROIs, there were significant difference between CK and GK metrics. However, for DTI metrics, the significant difference was detected only for NAWM regions for FA, MD, and RD metrics.

**Table 2:** The effect size of significant differences between low- and high-grade gliomas detected by CK and GK derived diffusion metrics (see Fig. 5)

Effect size	FA	MD	AD	RD	KA	MK	AK	RK
CK	0.99	1.21	1.11	1.23	0.11	1.56	1.47	1.52
GK	0.65	1.15	1.13	1.15	0.30	1.50	1.35	0.24

The effect size is estimated using the Cohen’s *d*

**Fig. 6:**
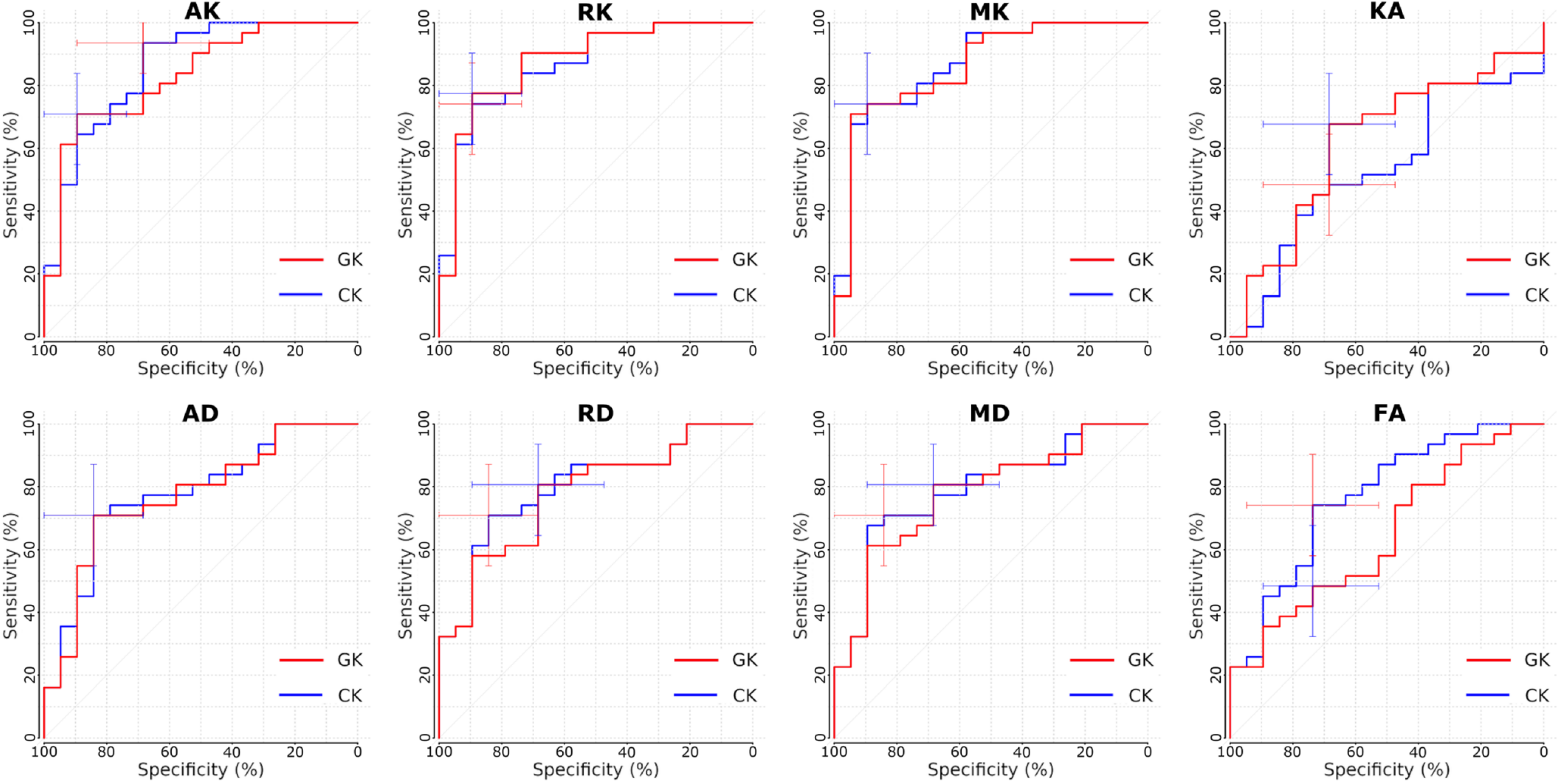
Receiver operating curves (ROC) for the conventional (CK) and generalised kurtosis (GK) approaches for differentiation of low- and high-grade gliomas. The error bars exhibits the detected sensitivity and specificity of both methods

It is of vital importance to perform a comparison between CK and GK approaches in terms of their sensitivity and specificity. These results are presented in Fig. 6. Both approaches demonstrated high rate of sensitivity and specificity for low and high glioma discrimination with negligible difference between CK and GK approaches. The estimated sensitivity, specificity, cutoff, and area under curves (AUC) are summarised in Table 3.

**Table 3:** Differentiation performance for diffusion metrics estimated by CK and GK approaches

	FA	MD	AD	RD	KA	MK	AK	RK
CK								
AUC	0.77	0.79	0.78	0.80	0.53	0.87	0.86	0.86
Cutoff	0.15	1.50	1.72	1.39	0.23	0.53	0.41	0.54
Sensitivity	0.74	0.71	0.71	0.71	0.48	0.74	0.93	0.74
Specificity	0.74	0.84	0.84	0.84	0.68	0.89	0.68	0.90
GK								
AUC	0.65	0.78	0.77	0.78	0.62	0.86	0.83	0.88
Cutoff	0.17	1.71	1.80	1.59	0.39	0.73	0.81	0.71
Sensitivity	0.48	0.80	0.71	0.81	0.68	0.74	0.71	0.77
Specificity	0.74	0.68	0.84	0.68	0.68	0.89	0.89	0.89

AUC of ROC (see Fig. 6), cutoff, sensitivity, and specificity values are presented

**Fig. 7:**
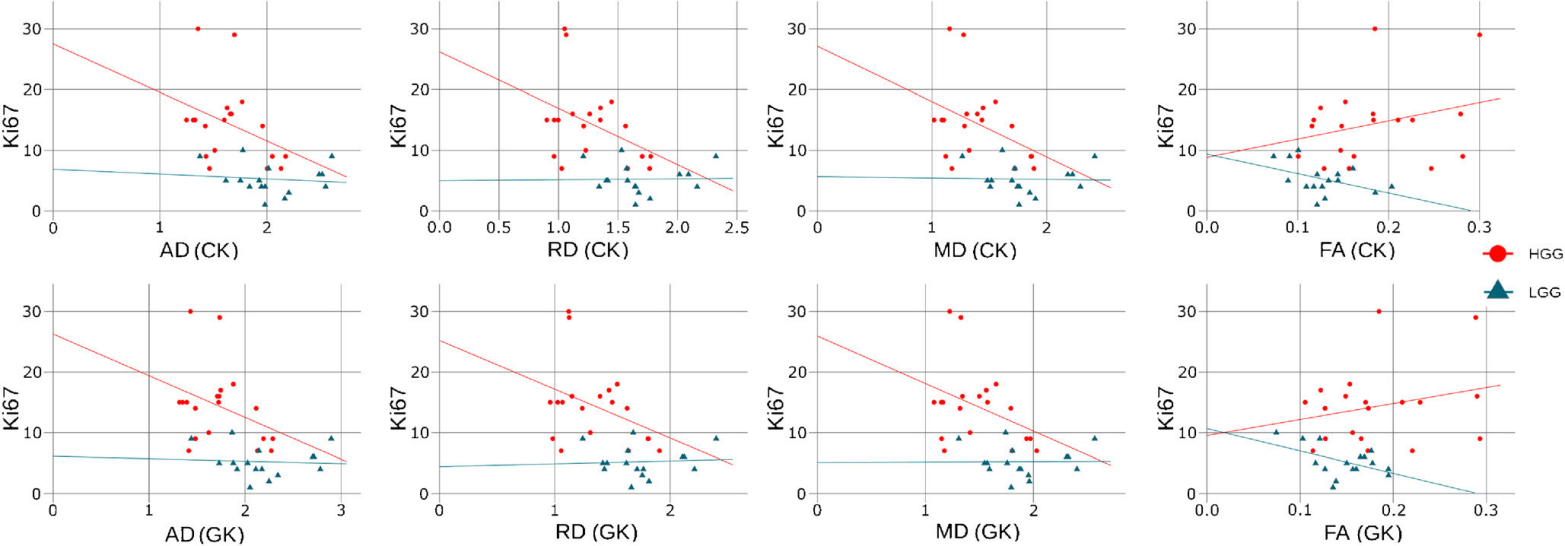
Correlation plots between Ki-67 and DTI metrics derived from CK and GK approaches. For each glioma grade group (low-grade LGG; high-grade HGG), we plotted the linear regression lines

**Fig. 8:**
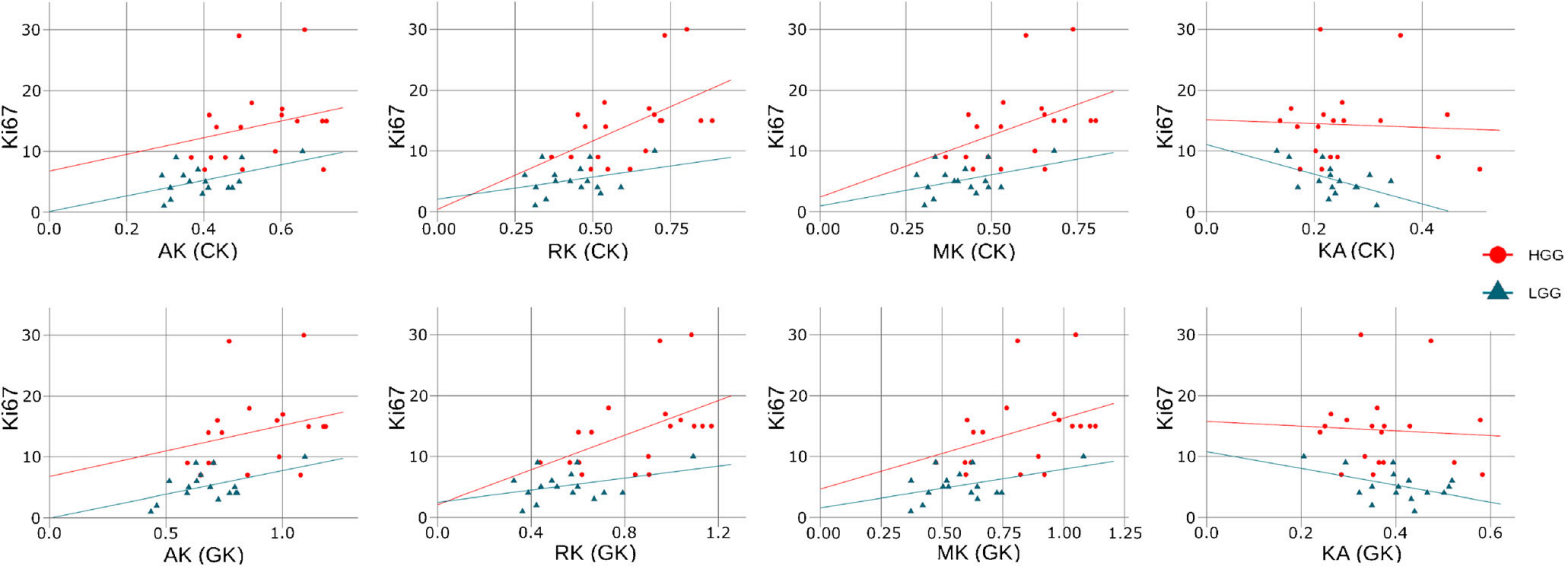
Correlation plots between Ki-67 and DKI metrics derived from CK and GK approaches. For each glioma grade group (low-grade LGG; high-grade HGG), we plotted the linear regression lines

In Figs. 7 and 8, we present correlations between Ki-67 and diffusion metrics estimated by CK and GK approaches for DTI and DKI, respectively. The correlations were estimated for all subjects and low- and high-grade subject groups separately. The Spearman’s correlation coefficients for diffusion metrics vs Ki-67 are summarised in Table 4. In Figs. 7 and 8, we plotted the linear regressions for low- and high-grade gliomas and performed a quantitative comparison for each approach using *cocor* function [33]. The significant difference (*p* < 0.05) between LGG and HGG correlations was found only for FA linear coefficients in both CK and GK approaches.

**Table 4:** The Spearman’s correlation coefficients *r* between Ki-67 and diffusion metrics estimated by CK and GK approaches

	FA	MD	AD	RD	KA	MK	AK	RK
*r* (all)								
CK	− 0.17	0.21	0.17	0.21	0.02	− 0.11	− 0.05	− 0.14
GK	− 0.19	0.22	0.17	0.23	0.01	− 0.07	− 0.03	− 0.08
*r* (HGG)								
CK	0.22	− 0.34	− 0.30	− 0.30	0.01	0.42	0.31	0.50
GK	0.09	− 0.29	− 0.25	− 0.29	− 0.12	0.43	0.40	0.58
*r* (LGG)								
CK	− 0.35	− 0.17	− 0.12	− 0.17	− 0.46	0.23	0.31	0.13
GK	− 0.33	− 0.13	− 0.16	− 0.07	− 0.33	0.21	0.24	0.16

**Fig. 9:**
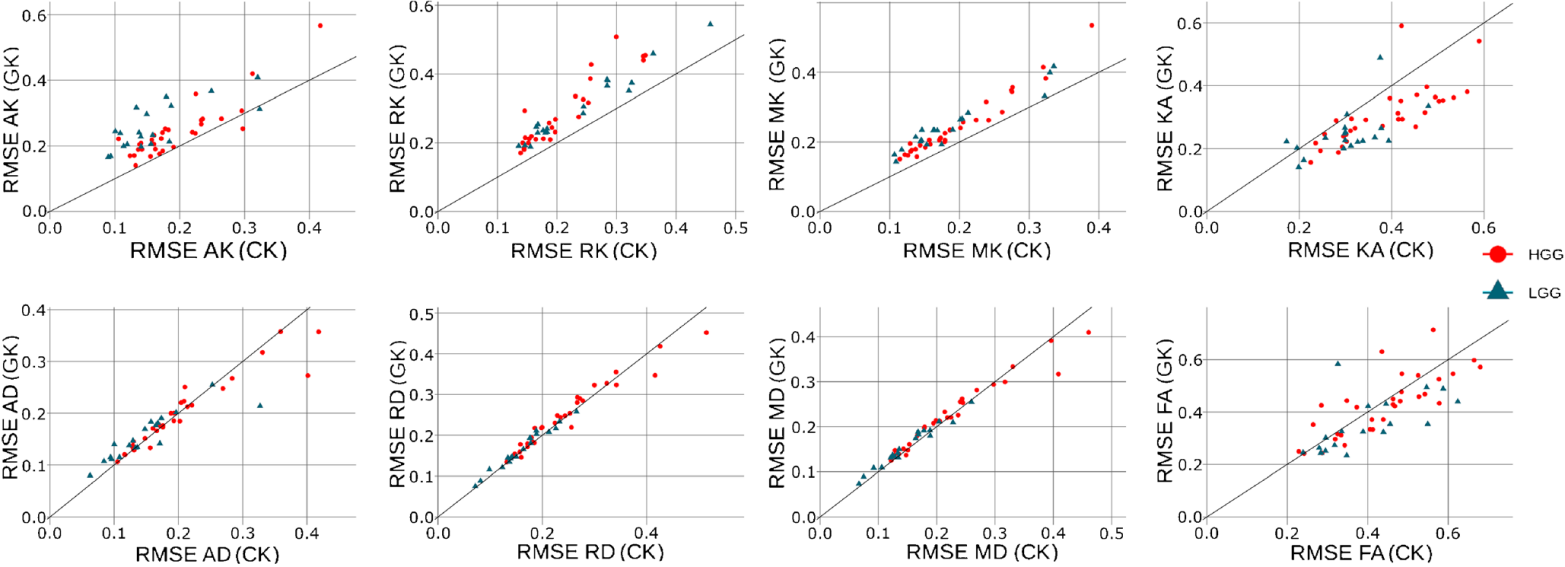
Scatterplots of root-mean-square estimators (RMSE) computed for CK and GK approaches. The RMSE rates were computed for the solid tumour regions only

Figure 9 shows a variability of the diffusion metrics in the solid tumour. The tumour heterogeneity was estimated by RMSE rate obtained for each DKI approach. In order to visualise the tumour heterogeneity, we used the scatterplots for each metrics. Interestingly, the heterogeneity derived from DTI metrics for both approaches does not have the significant difference, i.e. the RMSE points are close to the unity line (see Fig. 9). In turn, the RMSE rates for AK, RK, and MK are higher for GK approach, and the RMSE rate for KA is higher for CK approach. It is worth to note that the heterogeneity estimations obtained by GK approach show higher RMSE rates for both low- and high-grade gliomas.

## Discussion

In the present study, we estimated two diffusion imaging approaches based on the conventional and generalised kurtosis expansions. The scalar metrics based on GK approach demonstrated higher linear correlations with CK-derived metrics for high-grade glioma and comparable sensitivity and specificity in glioma differentiation between low- and high-grade gliomas. In turn, the scalar diffusion metrics based on GK approach exhibited brighter tumour and peritumoural oedema contrasts compared to healthy tissue allowing clinicians to better visualise tumour spreading in a healthy brain tissue environment. Moreover, the heterogeneity rates estimated by RMSE measure are higher in the case of MK, AK, and RK metrics derived from gDKI approach.

The tumour grade differentiation constitutes a challenge for many imaging techniques such as MRI, CT, or PET. This is due to the need of a quantitative parametrisation enabling both the assessment of damage of healthy brain tissue by aggressive growth of tumour and the degree of the malignancy of this process. This is particularly complicated due to multifarious forms of tumour manifestation, including cell swelling, microvasculature proliferation, tumour heterogeneity, and presence of necrosis. Thus, the diffusion MR imaging grants two levels of tumour assessment, that is, easy-to-interpret scalar maps and sensitivity of these maps to the micrometre scale of tissue changes. Therefore, diffusion kurtosis imaging offers an excellent tool for probing brain tumour, enabling estimation of the qualitative and quantitative differences between different glioma grades and their dynamics.

The difference between the conventional and generalised kurtosis estimations is defined by a fraction multiplier *α* and a quadratic kurtosis term in Eq. (2). It is interesting that dispersion in the distribution between DTI metrics (see Fig. 3) is quite low and does not depend on glioma grade. However, in the case of kurtosis scalar maps (see Fig. 4), in particular for MK and RK, there is a stronger value spreading in low grade glioma metric correlations. Such kind of distribution behaviour can indicate two different types of tissue organisation present at early stages of the glioma tumour: resting healthy brain tissue and highly heterogeneous tumour. As glioma malignancy increases, the heterogeneous tumour tissue becomes dominant due to aggressive growth which leads to higher correlations between CK and GK metrics, i.e. quadratic kurtosis term might drive this effect. We see that GK-derived MK and RK metrics are more sensitive to the tumour heterogeneity in low-grade glioma. Therefore, a variation in data dispersion between CK and GK metrics, in particular, in kurtosis metrics, might provide us with an additional information about glioma grades. Notably, this information is accessible only for voxel-wise assessment, where typical glioma grade differentiation is performed for region-averaged diffusion metrics.

This hypothesis is supported by significant differences between CK and GK metrics for kurtosis metrics in contrast to the DTI ones (see Fig. 5). For kurtosis metrics, the significant differences between CK and GK derived values were found in all regions: tumour, oedema, and NAWM. On the other hand, DTI metrics demonstrated significant differences only for normally appearing brain tissue, likely due to the different regions of the brain chosen as NAWM and strong age dependence of the patients. Thus, the quadratic kurtosis term strongly influences the kurtosis scalar metrics while it does not affect the DTI ones.

It is quite important to estimate general brain changes associated with tumour grades in accordance with healthy tissue. All DTI metrics (FA, MD, AD, and RD) demonstrated their potential as a glioma grade biomarker, except for FA maps derived from GK (see Tables 2 and 3). The values of MD, AD, and RD show lower diffusivities in high-grade glioma for both CK and GK approaches. In turn, diffusion metrics for both low- and high-grade gliomas demonstrated higher diffusivities in contrast to the NAWM values. This effect might be related to damage of healthy tissue as extra-cellular space increases accompanied by simultaneous microvascularisation process in the tumour tissue. Interestingly, low-grade glioma tissue retains higher diffusivities in contrast to high grade one, likely due to an increased cell proliferation rate in high-grade glioma, i.e. emergence of more cellular barriers limiting the water diffusion.

Kurtosis scalar maps exhibited the same trends as DTI metrics for low and high glioma differentiation and the contrast between the tumour and healthy tissue regions. Notably, KA did not reveal significant differences between low- and high-grade gliomas. Nonetheless, MK, AK, and RK metrics demonstrated lower kurtosis values in low-grade glioma in contrast to both high-grade glioma and healthy tissue (see Fig. 5).

We did not find significant differences for oedema regions for neither low- nor high-grade gliomas. At the same time, oedema metrics exhibited close range of kurtosis and DTI values with high-grade glioma, suggesting that the peritumoural tissue organisation is more complex than free water diffusion. This effect may be caused by the compression of the surrounding cells and resting microstructure of healthy tissue. However, this hypothesis should be verified and examined with different types of glioma spreading processes such as the level of cell infiltration that demands high-resolution diffusion measurements.

Our results showed the low correlations between proliferation rate Ki-67 and diffusion metrics (see Figs. 7 and 8) for all subjects. In the case of DTI metrics, we found that diffusion metrics obtained for high-grade gliomas exposed a moderate Ki-67 correlation rate in contrast to the metrics from low-grade gliomas. In turn, DKI metrics for both CK and GK approaches exhibited the high correlations with Ki-67 rate independently on the glioma grade. Encouraging, our findings of the Ki-67 correlations are in agreement with the previously published works [9, 34, 35].

As a limitation of the study, we should note not an optimal choice of adjusting variable *α*. A more accurate model fitted to the tumour microstructure [36] might increase feasibility of GK approach and its performance in the case of glioma grading. Additionally, advanced diffusion techniques including fast kurtosis [37], isotropic diffusion weighting [38–40], and multidimensional diffusion imaging [41, 42] might help clinicians to perform a robust, fast, and non-invasive glioma differentiation. We plan to implement and apply these approaches for research and patient treatment in the future.

In conclusion, the generalised diffusion kurtosis imaging presents an additional source of information enabling differentiation of low- and high-grade gliomas at the same level as the conventional DKI. The GK approach exhibited higher tissue contrast and, thus, offers more sensitive scalar maps to glioma tissue heterogeneity.
